# Development of Cognitive and Physical Exercise Systems, Clinical Recordings, Large-Scale Data Analytics, and Virtual Coaching for Heart Failure Patients: Protocol for the BioTechCOACH-ForALL Project

**DOI:** 10.2196/17714

**Published:** 2020-05-04

**Authors:** Antonis Billis, Niki Pandria, Sophia-Anastasia Mouratoglou, Evdokimos Konstantinidis, Panagiotis Bamidis

**Affiliations:** 1 Laboratory of Medical Physics Faculty of Health Sciences Aristotle University of Thessaloniki Thessaloniki Greece

**Keywords:** chronic heart failure, treatment adherence, exergames, e-coaching, adherence, electroencephalogram, wearable monitoring

## Abstract

**Background:**

Heart failure is a chronic disease affecting patient morbidity and mortality. Current guidelines for heart failure patient treatment are focused on improving their clinical status, functional capacity, and quality of life. However, these guidelines implement numerous instructions including medical treatment adherence, physical activity, and self-care management. The complexity of the therapeutic instructions makes them difficult to follow especially by older adults.

**Objective:**

The challenge of this project is to (1) measure real-life adherence to a regular physical exercise program and (2) attempt to influence older adult patients with heart failure toward embracing a more physically active self-care lifestyle.

**Methods:**

This research consists of two studies, including a lab experiment and a pragmatic evaluation of technology at patients’ homes. The lab experiment aims at exploring in an objective way (measuring neurophysiological responses to stimuli) patient engagement with different characteristics of virtual agents, while the home study is a 3-phase prospective study where the developed technology platform is tested by heart failure patients in their own home environments. Patients undergo evaluation of their physical activity and cognitive status using standard evaluation methods (6-minute walk test, questionnaires) and receive wearable devices to accurately measure everyday life activity levels (home study phases 1-3). During home study phases 2 and 3, exergames (serious games for physical exercise) to provide a physical exercise plan as a joyful activity are delivered to patients’ private households and e-coaching techniques are implemented in the final phase (home study phase 3) of the protocol, to influence patient attitudes toward a more healthy and recommended lifestyle.

**Results:**

The trial is still ongoing. Recruitment is ongoing, and the project has progressed for some participants through phase 2 of the home study. The sample size for both studies is 28 participants; 10 have already been included in the study, and both baseline clinical and patient-reported outcome data are retrieved. Phases 2 and 3 of the home pilot study are expected to be completed within 6 months.

**Conclusions:**

The main challenge of the project is the change of attitude of older age heart failure patients through an e-coaching system. Given the adoption of a cocreation and living lab approach and the main objective for real-life evaluation, the project is ready to react to any collected feedback, even during the implementation of the research plan. Clinical assessment and objective evaluation are expected to provide all required information for reliable findings.

**Trial Registration:**

ClinicalTrials.gov NCT03877328; https://clinicaltrials.gov/ct2/show/NCT03877328

**International Registered Report Identifier (IRRID):**

DERR1-10.2196/17714

## Introduction

### Background

Heart failure (HF) is a clinical syndrome affecting more than 15 million people in Europe and more than 30 million patients worldwide [1]. Despite advances in its management, prevalence of the disease is expected to increase mainly due to the aging of population, making the disease a constantly worsening global problem. Studies have proven the effectiveness of rehabilitation programs consisting of systematic physical exercise and self-care in HF patients [2-6]. Among them, regular aerobic exercise is recommended in HF patients in order to improve their functional capacity and symptoms [7]. However, changing lifestyle and engaging self-care, especially in older adults, is a difficult task and a barrier to engaging older adults with HF in regular physical exercise. Although therapeutic interventions seem to reduce admission rates for patients with HF, effective management of the disease remains a contemporary challenge. Current guidelines for HF patients emphasize improvement of clinical status, functional capacity, and quality of life, implementing complex regimens of multiple self-care behaviors (systematic exercise, fluid and sodium restriction, adherence to medical therapy, and close monitoring of the development of disease symptoms, etc) to medical treatment [7]. The complexity of the instructions and necessity of lifestyle modifications in combination with possible comorbidities and cognitive decline make the guidelines difficult to follow, especially in older adults [3].

The challenge of this project is to measure real-life adherence to a regular physical exercise program and attempt to influence older age patients with HF toward being more active. To do so, BioTechCOACH-ForALL uses wearable devices to measure activity levels, exergames (serious games for physical exercise) to deliver a physical exercise plan as a joyful activity, and e-coaching techniques to influence patient attitudes toward HF self-care and more healthy lifestyles.

### Protocol Concept and Rationale

Cardiovascular disease is common among older adults. In developed countries, prevalence of HF in adult population is 1% to 2%, rising up to more than 10% in people over 70 years old [1]. Late complications of the disease and comorbidities such as coronary artery disease, systemic arterial hypertension, diabetes mellitus, history of stroke, anemia, dementia, kidney dysfunction, lung disease, and obesity contribute to the burden of hospitalizations and mortality [7] and are targets of treatment.

According to current guidelines, the goals of treatment in patients with HF are to improve clinical status, functional capacity, and quality of life. Although these are surrogate markers of treatment success, the need for reduction of hospitalizations and mortality is also clearly indicated [7]. Furthermore, lifestyle modifications like implementing healthy nutrition and systematic exercise and smoking cessation as well as self-care including but not limited to monitoring body weight and avoiding excessive fluid and salt intake are deemed necessary [2]. Although the disease may sometimes be life-limiting, exercise is encouraged in all clinically stable patients with HF, and regular aerobic exercise is recommended in HF patients (class IA according to current guidelines [7]) in order to improve their functional capacity and symptoms [8]. Various exercise rehabilitation programs have been used in HF, consisting of bicycle ergometer training, dumbbell training using low weight (<1 kilogram), respiratory training, and walking about 5 times per week. Fatigue severity, 6-minute walking distance, respiratory function, and quality of life are improved via increased physical activity of HF patients [7,8]. To this extent, close monitoring of daily mobility and sedentary patterns with wearables and tailor-made e-coaching systems based on activity profiles and routines of HF patients, implemented in everyday life, may promote exercise integration by making it challenging for the patient, who may set their own realistic activity goals.

Patients with HF should also follow their medical pharmacotherapy, a task that might be difficult because of cognitive disorders and coexisting comorbidities leading to polypharmacy, often obligating a caregiver to help them with this daily task. Despite clear evidence of the benefits of adherence to medical therapy to the rates of morbidity and mortality and number of cardiovascular-related emergency department visits in HF, rates of patient adherence to medical and supportive therapy (the extent to which a patient’s behavior with regard to medication intake or lifestyle changes is consistent with therapeutic recommendations) vary significantly, fluctuating between 10% and 98% [7,9,10]. On the other hand, there is more clear evidence on the nonadherence (noncompliance to treatment) of patients, which was found to be almost 25% in the general population, with men and women showing the same rates of noncompliance to treatment. It has been shown that adherence to HF medication is related to patient institutionalization (including hospitalizations and nursing home visits) [11], while patient self-care (eg, self-care management; self-care maintenance; sodium, fluid, and alcohol intake restriction; physical activity; smoking cessation; monitoring signs and symptom; and keeping up follow-up appointments) is positively related to the length of time since the patient was diagnosed with the disease [12].

Given the constantly increasing number of patients with HF, patients’ demands on health care services are expected to increase greatly in the coming years. The need for more innovative and cost-effective treatment strategies led to studies of electronic health (eHealth) programs showing promising results in patients with HF [13-15]. These studies increased political and clinical attention to eHealth strategies as a mean of improving outcomes in patients with HF. However, the role of eHealth systems in the management of patients with HF and in particular in the practical implementation of adherence (eg, by promoting packages of measures concerning medical treatment and active living, patient education and active participation in the context of shared decision making to develop realistic expectations of their own disease course, and being active and adopting individual responsibility) is an emerging field of high scientific interest.

### Designing an eHealth System Using Virtual Coaches

Designing an eHealth system to promote self-care of patients remains challenging. User engagement constitutes a key component for considering technologies successful. O’Brien and Toms [16] defined user engagement as follows: “Engagement is a category of user experience characterized by attributes of challenge, positive affect, endurability, aesthetic and sensory appeal, attention, feedback, variety/novelty, interactivity, and perceived user control.” At the same time, the presence of human social models has been shown to affect attitudes, beliefs, and behaviors of users [17,18]. Moreover, anthropomorphic agents could have impact on cognitive functioning and exert social influence comparable to that of humans [16] while also promoting motivational characteristics such as self-efficacy and attitude change [19]. Furthermore, the use of pedagogical or virtual agents could facilitate learning [20]. Therefore, using a virtual coach with specific characteristics could possibly increase both technology acceptance and user engagement.

The influence of virtual agents on users could vary depending on different characteristics such as availability, communication skills, believability, functionality, and customizability in appearance [20]. In that sense, social models were found to be more effective as they resemble the observer or a projected ideal virtual self of the observer [19]. Existing evidence on learning showed that agents who had similar characteristics to trainees, with respect to appearance-related traits such as age and race/ethnicity, could be more influential [19]. However, prior expectations and stereotypes could influence the desired outcomes [20]. Additionally, perception of self in a virtual environment affects task-related, verbal, and nonverbal behaviors [21]. In line with that, researchers introduced the Proteus effect, which describes the condition where people conform to their avatar representation regardless of how other people perceive them [22]. Another study showed that a physically similar avatar to the observer could affect the emotional valence and arousal more than a neutral one. Additionally, the induced emotional states were more intense than those from neutral avatars [23]. Therefore, appearance is considered to be an important attribute while designing a virtual agent. Another important design element was shown to be that the agent stays within the field of vision of the participants [24,25].

The way that a virtual agent uses to communicate is another component for customization. Social presence consists of verbal and nonverbal cues. However, agent communication through voice has been found to be more beneficial than text. More precisely, the use of a human-like voice could enhance social presence and interaction with technology [16]. Moreover, facial expressions and deictic gestures are considered to be crucial for promoting learning-related outcomes. However, the large-scale study of Baylor and Kim [19] stressed that facial expressions—but not gestures—seem to enhance focus on the motivational message delivered by the virtual agent.

The evaluation of technology acceptance and user engagement in an explicit way remains a challenge. Fairclough et al [26] defined user engagement in a task in terms of cognitive activity (mental effort), motivation (approach or avoidance), and affective state (positive, negative), and they associated the user engagement’s components with psychophysiological measurements. Revisiting the literature, they found that increased theta activity in frontocentral sites along with decreased alpha activity in occipital sites was associated with higher mental effort due to working mental load. Pupil dilation was observed to be greater when complex cognitive processing is performed.

On the other hand, motivation and emotional experience (affect) were correlated to frontal asymmetry. More precisely, greater levels of left frontal activity were associated with positive emotions and motivational approach, whereas higher right frontal activity was linked to negative emotions and motivational avoidance. Other biomarkers of motivation were considered to be sympathetic nervous system indices, such as systolic blood pressure. In another study, user approval of an online avatar was explored by means of skin conductance, heart rate, and respiration. Results indicated that higher respirations were positively correlated with the degree of agent approval [27].

Peters et al [24] proposed user attention as another metric of human-agent interaction. They modeled user attention using three components: gaze detection; neurophysiological analysis; and an attention representation module for storage, integration, and interpretation of attention information.

### Study Objectives

High rates of noncompliance to treatment plan indicate the need for developing sustainable solutions to support and enhance the self-care of HF patients. BioTechCOACH-ForALL, implemented within the framework of the operational program Human Resource Development, Education, and Lifelong Learning and cofunded by the European Social Fund and national resources, investigates and researches a potential response to this challenge.

The main goals of the project are as follows:

- Extension of previous experience in developing and applying innovative systems for physical training of elderly (webFitForAll [13]), in living labs or even at their home [28], encouraging physical exercise and promoting independent living. In addition, the e-coach platform will be enriched by a decision support system (smart algorithms that will personalize the interaction of the e-coach with patients) based on analysis and collection of interaction data. Commercial nonintrusive sensors will collect activity data in order to capture daily activity patterns [29] and activity volume. Daily activity patterns will be used to track their daily activity level regarding the doctor’s recommendation and readjust e-coaching system parameters (home study phases 1 and 2)
- Development of an e-coaching system (home study phase 3) based on neuroscience evidence (lab study), incorporating exergaming [30] and remote health monitoring [29] techniques
- Patient engagement with different user interface interaction means, such as virtual projected coaches with different characteristics (presence/absence of medical uniform, gender, age) will be explored by means of electroencephalogram (EEG) and analyzing various biosignals such as heart rate, electrodermal activity, external body temperature, and eye gaze tracking

## Methods

### Overview

Two studies will be performed. The first study aims to optimize patient acceptance of the delivered technology solution and in particular the e-coaching virtual agent by evaluating different design characteristics introducing a novel lab experiment and objective measurement of patient engagement with the use of EEG and biosignal markers, while the second (at home) study aims to introduce the technologies and interventions (exergaming and e-coaching) to the HF patients’ daily routine. Results and findings of the first study will drive the design of the e-coaching intervention that will be applied in the third phase of the second study.

### Laboratory Study

The rationale of EEG study is to explicitly capture the way patients perceive images of virtual agents by recording different biosignals. In that sense, analysis of multichannel event-related EEG data could reveal differences in spatial distribution and temporal sequencing of neural activity between different conditions such as presence versus absence of medical uniform, old versus young, and female versus male [21,31,32]. Moreover, other biosignals such as electrodermal activity, external body temperature, heart rate, and eye gaze tracking have already been applied to evaluate affective and cognitive impact of projected stimuli [24,33]. As such, HF patients will undergo a 2-part experimental procedure in which various biosignals will be recorded via EEG, E4 smartwatch (Empatica Inc), and GP3 eye tracker device (Gazepoint). In both parts of the experimental procedure, participants were instructed to freely observe the images. In the first part, participants will passively observe images of virtual agents, presented on screen as stimuli having different appearance characteristics such as age, gender, and presence/absence of medical uniform. In the second part, stimuli presented to participants will be pairs of virtual agents followed by a fixation cross on black background. The pair of agents differs regarding the presence/absence of medical uniform but preserves all other characteristics (age, gender).

### At-Home Study Design

#### General Design

BioTechCOACH-ForALL home study is a prospective, multiple baseline across subjects, nonrandomized, single-arm, single-center study following a within-subject design to assess the feasibility and efficacy of the Virtual Coach Program in older age patients with HF. The study will be delivered in three phases, each of them fulfilling a different scope. All participants will go through all study phases. Each phase will allow participants to familiarize themselves with the delivered technology of that phase. A multiple baseline approach will be followed so that the effects of each phase are as isolated to the previous phases as possible, allowing for effect comparison among them. Clinical and quality of life assessment and exercise behavior and attitude will be measured repeatedly in both the baseline phase and the two intervention phases. This way any cause-effect relationships among the intervention and patient outcome measures will be demonstrated.

The study conforms to the ethical guidelines of the 1975 Declaration of Helsinki, all participants will sign informed consent, and the study protocol has been approved by the bioethics committee of the School of Medicine of the Aristotle University of Thessaloniki (Protocol No. 1.45/21.11.2018) and registered at ClinicalTrials.gov [NCT03877328]. The study protocol is structured in a manner that incorporates three different phases, coming one after the other. Each patient will enter the study in the phase 1 and complete their participation in phase 3.

#### Phase 1

Phase 1 introduces the objective measurement technology, the wearable monitoring device. This technology will be running throughout the project’s lifetime and will provide objective information on patient activity. This information, along with the doctor’s baseline and intermediate assessment, will be used as an indicator of the effectiveness of phases 2 and 3.

#### Phase 2

Phase 2 introduces a joyful way of exercising, allowing patients to exercise in the comfort of their homes. Patients will be assigned a structured recommended schedule with a goal of 3 sessions per week for a total of 36 sessions in 3 months. Frequency and intensity of the training program are indicated by patient functional capacity. All patients will undergo a 6-minute walk test, and their performance will be used for determination of the exercise program. By protocol, patients performing more than 500 meters in the 6-minute walk test at baseline will be prescribed a more intense exercise program in terms of the number of exercise repetitions. The exercise protocol offers 50 minutes of exercise implementing aerobic and resistance endurance exercises including upper and lower extremities. Regular blood pressure and heart rate monitoring will be performed manually by the patient with the use of dedicated devices in time intervals specified in the protocol. Exercises will be implemented as fun, full-body interactions, processed and recognized through a depth-camera–based sensor and computer vision and translated into computer game actions and scenarios integrated within a Web application [14].

#### Phase 3

Phase 3 introduces the coaching aspects, where exergames are introduced and delivered through home surface projection, apart from the personalized recommendations and suggestions (designed by the doctor).

### Patient Population

Both studies can fulfill their objectives only if appropriate subjects are enrolled. The following eligibility criteria are designed to select subjects. These criteria must be met before a subject is assigned to the study. Subject eligibility should be reviewed and documented by a qualified member of the investigators’ study team before subjects are included in the study. All patients included in the study protocol will undergo all three home study phases, in addition to the EEG experiment in the lab. For the latter phase, a percentage of patients, without the need to proceed with the whole study protocol, will be recruited.

### Selection Criteria

Subjects must meet all of the inclusion criteria to be eligible for the enrollment:

- Male and female patients aged over 55 years with HF of any etiology, with either reduced or preserved ejection fraction, diagnosed according to international guidelines [7]
- Must be in New York Heart Association (NYHA) functional class II-IV
- Must be in stable clinical condition and on stable medical treatment for the underlying disease for at least 3 months prior to inclusion to the study
- Must be willing and able to comply with scheduled visits, treatment plan, and trial procedures
- Must provide personally signed and dated informed consent document indicating that the subject has been informed of all pertinent aspects of the study

Subjects presenting with any of the following will be excluded from the study:

- Unstable disease with evidence of decompensation, recent hospitalization, or undergoing investigation for clinical deterioration
- Recent history of chest pain, palpitations, light-headedness, dizziness, or syncope on exertion
- Contraindications to physical activity or with physical obstruction to perform the prescribed training program (eg, patient uses wheelchair)
- Any severe acute or chronic medical or psychiatric condition that may increase the risk associated with trial participation or interfere with interpretation of trial results
- Investigational site staff members directly involved in the conduct of the trial and their family members; site staff members otherwise supervised by the investigator
- Participating in any other experimental studies
- Not willing to provide signed informed consent

### Materials and Technologies

**Figure 1:**
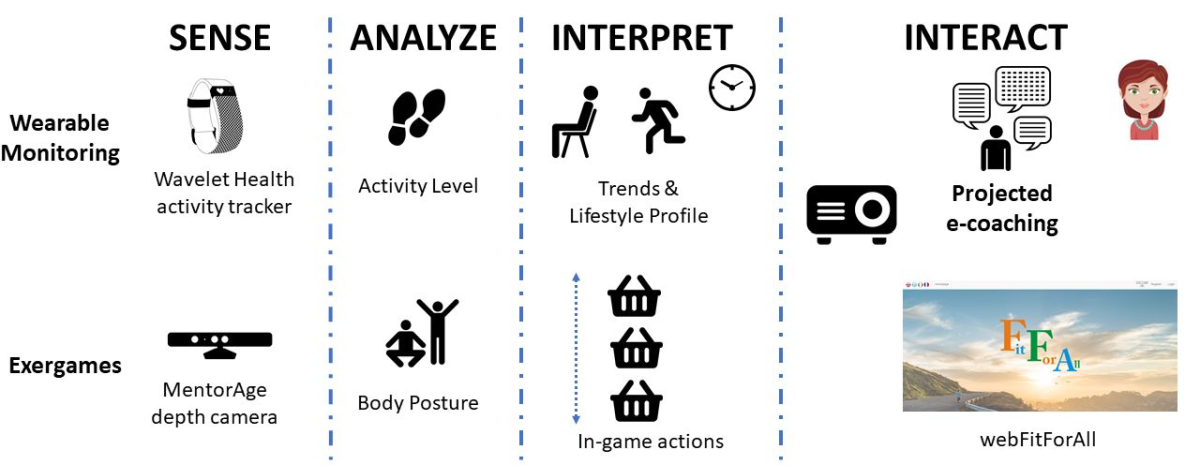
Protocol phases and technology introduced in each phase.

The technologies to be used for BioTechCOACH-ForALL along with their scope are presented in Figure 1 and include wearable continuous monitoring and lifestyle patterns discovery, exergames, and projected, smart e-coaching.

#### Wearable Monitoring

The wristband monitoring device (Wavelet Health) [34] includes a clinical-grade (red plus infrared) photoplethysmogram sensor along with accelerometer and gyroscope and can collect continuous physiological and activity data processed using robust algorithms. Actigraphy capture rates spanning from 1 Hz to 20 Hz, while light sensor capture rate can be either 43 Hz or 86 Hz. To balance energy consumption, light sensor capturing is enabled in cycles. This means it does not measure all the time, but it remains idle for some time and collects a single averaged measurement over the remaining time of the cycle.

Computed features include steps, calories, beats per minute, heart rate variability, SpO_2_, breathing rate, sleep staging (awake, light, deep), and total sleep time. These fitness and lifestyle analytics will be calculated both as intraday and interday time series so as to form different patterns within a day (allowing the system to understand daily habits) and trends in specific time periods (to quantify health and lifestyle changes). Different detailed levels of information will be explored like per minute or hour. Active time periods against sedentary moments will also be used as a way to explore patients’ activity habits during the day.

Collected information will be used as objective, real-life measurements for evaluation of different interventions compared with the baseline (activity levels as proxy indicator for active lifestyle habits) and as a way to personalize different parameters of the intervention (eg, time of the day or weekdays to suggest that patients perform exercise regimes).

#### Exergames Promoting Physical Exercise

In order to deliver a structured protocol of physical exercise to HF patients, a computerized intervention will be developed in the form of exergames as part of home study phases 2 and 3.

**Figure 2:**
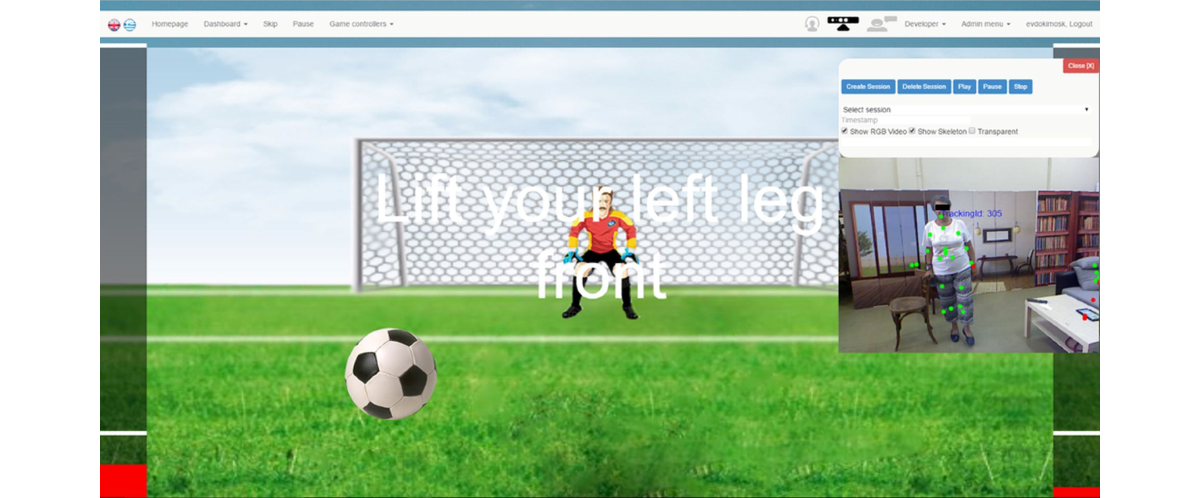
webFitForAll interface.

An existing exergame platform, webFitForAll [14], incorporates physical exercises recommended by the American College of Sports Medicine and American Heart Association [29] focusing on upper and lower strength, stretching and flexibility, and aerobic exercise, will be used for delivery of the tailor-made physical exercise program. An example of the webFitForAll interface is presented in Figure 2.

Adherence to the right execution of exercise is feasible by comparing several predefined parameters concerning the movements of patients. Joint angles are continuously monitored based on the tracking of body skeleton joints having as inputs the MentorAge device (Nively SAS) embedded RGB and infrared cameras, thus providing smart feedback to patients with respect to successful execution of the exercises. This mechanism ensures correct administration of the protocol and adherence of the patient to the instructions. webFitForAll allows patients to track their progress by evaluating their in-game performance with a single score. Motivational messages are delivered at the end of a game and the end of a training session to keep patients engaged with the intervention.

#### e-Coaching

This is the most important part of the solution (introduced at the last phase of the home study) since it integrates all previous components and their respective information while being the main patient-system interaction point. Daily recommendations about activity and patient self-management together with a virtual coach that will be selected based on findings of the neurophysiological study will be projected on a predefined surface in the patient’s home. The type of messages and time of delivery will be chosen/scheduled based on the personal profile of the patient (eg, nocturnal patterns of activity). Leaving the home environment intact is considered key to increase the acceptance of the e-coaching system by the patient. Therefore, any information visualization will happen only at predefined moments and will disappear instantly, by simply turning the projector off.

An added value of the study is personalization of the e-coaching system by means of evaluating the virtual agent characteristics using EEG, eye gaze tracking, and other physiological measurements.

### Procedures

Apart from the home study, a study in laboratory settings will be conducted to explore the design elements of the virtual agent that will be part of the e-coaching technology. Participants will undergo a 2-part experimental procedure in order to investigate the impact of different appearance-related characteristics of virtual coaches on user engagement, set up as follows: the study takes place in a magnetically shielded, sound attenuated, and dimly illuminated room hosted in the Laboratory of Medical Physics. EEG recordings are performed by means of a 128-channel EEG recording system (Nihon Kohden Corp) and a sponge-based passive electrode system (R-Net cap, Brain Products GmbH) applying the international 10-20 positioning system. Participants are comfortably seated in an armchair in front of a 23.5-inch computer monitor at a distance of 75 cm.

**Figure 3:**
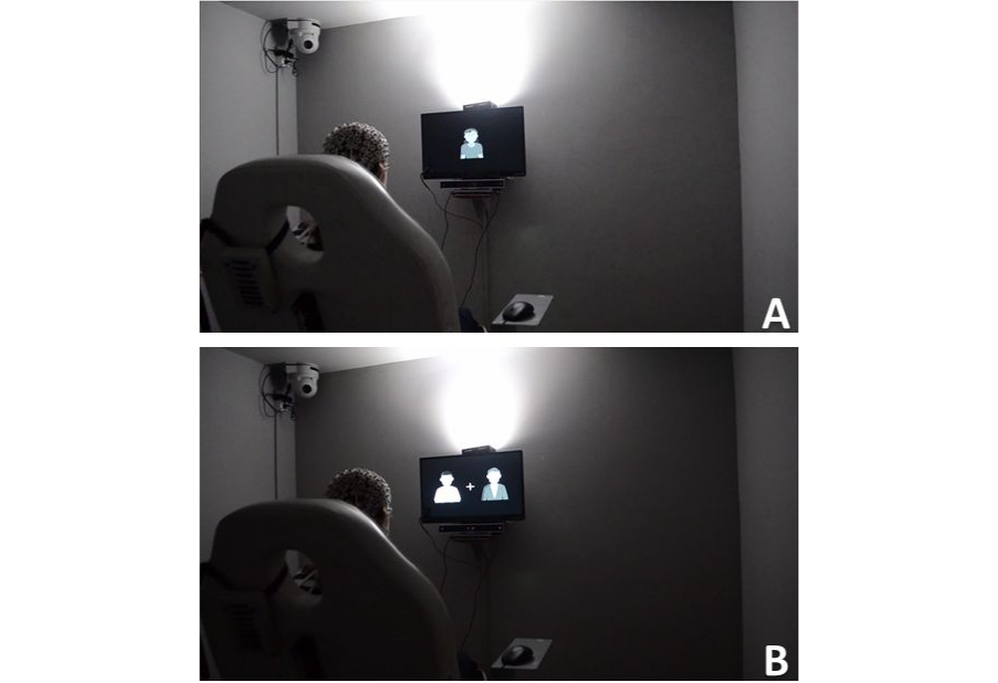
Electroencephalogram (EEG) study protocol. (A) Patient undergoes EEG while passively viewing single virtual agents. (B) Patient undergoes EEG while passively viewing combinations of virtual agents.

In the first part, the HF patients initially undergo EEG recordings during resting state with eyes closed (5 minutes). Participants then passively view 32 agents presented on screen as stimuli in a random order, grouped with respect to their appearance characteristics such as age, gender, and presence/absence of medical uniform (Figure 3A). During each trial, the stimulus (image of a virtual agent, height 6.22 cm, width 4.57 cm) is presented for 2000 ms in the center of the screen followed by a 2000 ms interstimuli period during which a black screen with a fixation cross is displayed. Each participant completes 256 trials (128 trials displaying images of virtual agents, 128 trials displaying black screen with fixation cross). In the second part, the stimuli presented to the participants during each trial consist of pairs of virtual agents and a fixation cross between them. The pair of agents differ regarding the presence/absence of medical uniform but are similar with regard to all other characteristics (age, gender; Figure 3B). Each stimulus appears for a duration of 2000 ms followed by an interstimuli period of 2000 ms, during which a black screen with a fixation cross is displayed in the center of the screen. The overall number of trials in this second part is 512 (256 trials displaying pairs of virtual agents, 256 trials displaying black screen with fixation cross) [35]. In both parts, participants are instructed to freely observe the images of virtual agents. During the EEG study, electrodermal activity, blood volume pulse, external body temperature, and eye gaze tracking are recorded for each participant. Biosignals will be collected by means of an E4 smartwatch (Empatica Inc), while eye gaze tracking will be performed using a GP3 eye tracker (Gazepoint).

### Intervention Setting

The first phase of the protocol will start in the 1st Department of Cardiology, AHEPA University Hospital of Thessaloniki, where all clinical assessments (6-minute walk test, questionnaires, clinical assessment, etc) will be completed, and it will continue in a dedicated area in the Laboratory of Medical Physics in the Faculty of Health Sciences of the Aristotle University of Thessaloniki, where patients will undergo the lab study/protocol. After the lab study, some of the patients will be included in the second study situated in their own homes, where they will keep performing their everyday activities. The physical training and e-coaching interventions (phases 2 and 3) will take place at patients’ homes as well.

### Patient Recruitment

According to the protocol, signed informed consent will be obtained by each participant at the baseline visit. It is the investigator’s responsibility to ensure that each study subject is fully informed about the nature and objectives of the study and possible risks associated with participation. The investigator will obtain written informed consent from each subject before any study-specific activity is performed. The investigator will retain the original of each subject’s signed consent document.

### Baseline, Intermediate, and Follow-Up Measurements

Brain Electrical Source Analysis software version 6.0 (BESA GmbH) will be used for data preprocessing. Visual inspection of the recordings will be performed to detect bad channels that will be interpolated using an interpolation algorithm of BESA software. The signal will be band-filtered at 1-30 Hz and a notch filter will be also applied.

Dimensionality of the data will be diminished by using principal component analysis, and an extended independent component analysis [36] will be performed. The reconstructed dataset will then be visually inspected. Subsequently, epochs will be averaged for different stimuli conditions (eg, female, male, old, young, doctors, peers). The randomization graphical user interface (Ragu toolbox [37]) will be used for statistically analyzing the multichannel event-related EEG data. More precisely, the total strength of scalp field differences will be estimated by means of global field power [38], and total count of significant time intervals [39] will be identified by running topographic analysis of variance. Afterward, cortical current density reconstruction will be calculated by low-resolution electromagnetic tomography [40] using BESA software in time intervals that will be derived by the aforementioned analysis.

Statistical parametric mapping will be applied for reslicing and statistical comparison of the current density reconstruction images exported by the BESA software between conditions using the SwE toolbox that applies the sandwich estimator method described by Guillaume et al [41], allowing analysis of longitudinal and repeated measures data. Other biosignals will be compared between conditions (young vs old, female vs male, doctors vs peers) after extracting the grand average values for each condition.

### Clinical Assessment

Patients will be clinically evaluated before entering each of the two studies and on the initiation of each protocol phase (meaning before entering phase 1, phase 2, and phase 3, and at the end of phase 3, which will mark the end of study), completing 4 on-site clinical visits for the impact of each intervention to be assessed.

On the baseline, intermediate, and follow-up assessments, patient clinical condition, quality of life, and health-related costs will be considered. More specifically, blood pressure, heart rate, blood oxygen saturation, and body weight will be measured for the clinical assessment. To assess physical status, the 6-minute walk test and NYHA functional class will be used for exercise intolerance. Patient-related outcomes to be used include the Beck Depression Inventory [42] and the Dukes questionnaire [43]. As for the evaluation of quality of life, the Short Form Health Survey questionnaire [44,45] will be employed. Self-efficacy for exercise behavior scale will be used for evaluation of changes in patient perspectives on exercise [46]. Finally, for health-related costs, the effect on number of hospital admissions along with the effect on health care use (number of primary and secondary care contacts, social care contacts, relevant medication use) will be calculated during all three phases.

Real-world data will be collected continuously (across all phases of the home study) to assess several aspects of the interventions planned. Continuously measured activity levels expressed in daily steps taken by the patient will be compared across the different study phases. Real-life adherence of the HF patients to the proposed intervention will be measured in terms of attendance at the webFitForAll platform using wearable heart rate monitoring data as well as online activity logs and telephone and clinic follow-up. Real-world adherence will be compared within-subjects for the second and third phases of the study. Use analytics (virtual coach used, content and delivery time of messages/recommendations) of the e-coaching system and juxtaposed relevant outcomes (such as activity levels collected by the activity tracker and adherence to the webFitForAll training program given any system logs) will be routinely collected. Patients adherence will be evaluated by measuring attendance at the webFitForAll and heart rate monitoring data as well as by activity logs and telephone and clinic follow-up.

### Statistical Analysis

Continuous variables with normal distribution will be reported as mean and standard deviation, while those with nonnormal distribution as median and interquartile range. Categorical variables will be expressed as frequencies and percentages. Continuously data collected will be explored for normality assumption by means of a Shapiro-Wilk test to calculate the appropriate descriptive statistics [47].

Repeated measured analysis of variance or Friedman test will be used to assess changes on continuous data with normal and nonnormal distribution, respectively, between baseline, follow-up, and end-of-study visits. Possible associations between variables will be investigated using Pearson or Spearman correlation coefficients. We estimated sample size conducting power analysis using G*Power software (version 3.1). We performed repeated measures analysis of variance (3 time conditions) using 80% power, a medium effect size of 0.25, and significance level of 5%. The sample size was estimated to be 28 participants. One-third of this patient population that will agree to proceed to home study (phases 1 through 3) will enter the next steps of study protocol [48]. All statistical analyses will be performed using SPSS Statistics version 23.0 (IBM Corp) or R for Windows version 3.1.3 (R Foundation for Statistical Computing).

### Technical Solution Deployment and Release

**Figure 4:**
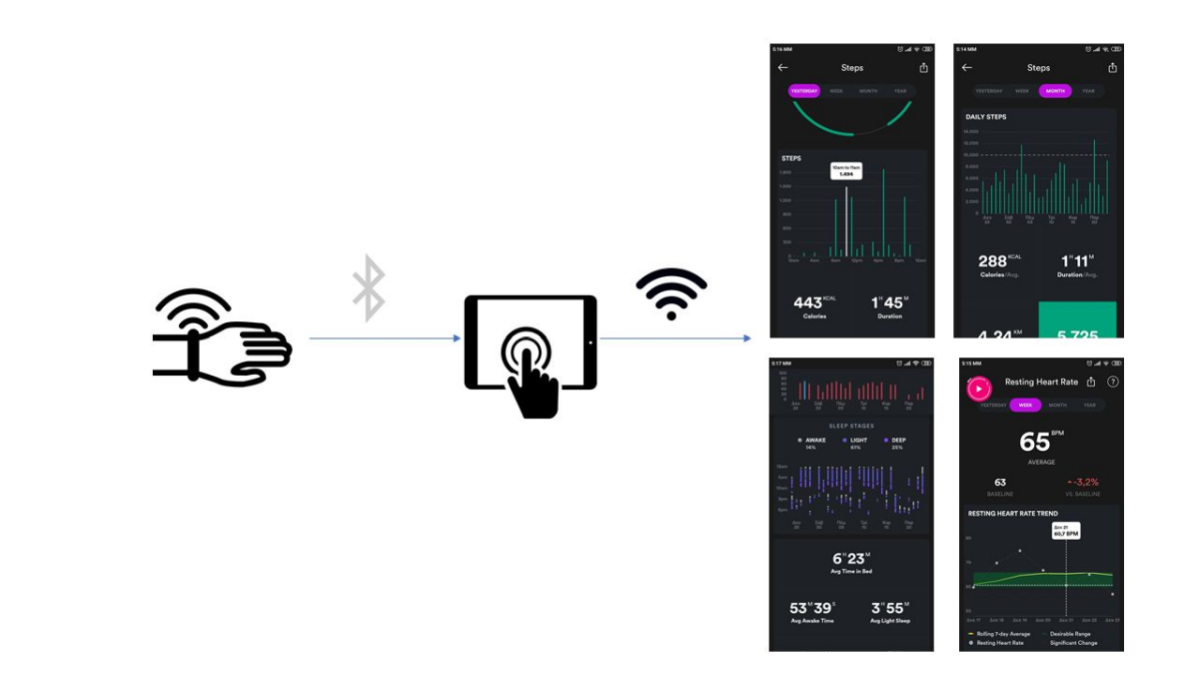
Wearable devices used to evaluate patient protocol adherence.

Given that the project relies on deploying the releasing of technology and devices, special attention has been paid on the planning of the releases. To be more specific, the off-the-shelf activity trackers (Wavelet Health wristbands) are delivered first (home study phase 1). The activity tracker will be set up to synchronize its raw data to the server through an app installed on the smartphones of patients. Patients not owning a smart device compatible with the provided software and hardware will be provided one. Synchronization will be done periodically through the day without any need for the patients to interact with the app installed in their phones. The app will synchronize in the background all gathered raw data, which will then be analyzed on the server to derive all meaningful features. Figure 4 presents a schematic approach of the wearable monitoring attached in the protocol.

Next, the webFitForAll platform along with the MentorAge device, which embeds a depth and RGB image sensor, will be introduced to patients in lab settings in order to train them on how to interact with it. MentorAge will be installed to monitor body movements by extracting and analyzing the body’s skeleton and silhouette. MentorAge operates on Android OS and can support any graphics, thus being able to act as an end-user terminal. To do so, a mini projector will be plugged in and set up by team members in MentorAge to display the e-coaching output to any home predefined surface, taking into account unobtrusiveness and patient acceptability. After an introductory session, patients will have the exergaming platform installed at their homes (home study phases 2 and 3). Safety precautions and instructions on how to perform exercises will be given by a nurse.

## Results

Patient recruitment is completed, and the project has progressed through phase 2. In total, 10 patients have been included in the study, and baseline clinical and patient-reported outcome data are retrieved. All participants included were male with a mean age of 63.60 (SD 8.78) years suffering from HF due to coronary heart disease (8/10, 80%) or arterial hypertension (1/10, 10%) while 1 (10%) patient suffers from dilative HF. The majority of participants (7/10, 70%) reported active employment status. The most common comorbidity of participants was diabetes mellitus whereas other conditions mentioned were arterial hypertension, chronic kidney disease, and chronic obstructive pulmonary disease. In terms of their social status, 9 of 10 participants live with their family, and 80% (8/10) of participants were married. The majority of patients had preserved functional capacity, classified as NYHA class II (6/10, 60%). The mean distance walked on the 6-minute walk test was 443.00 (SD 99.78) meters. Phases 2 and 3 of the pilot study are expected to be completed within 6 months.

## Discussion

### Expected Outputs and Potential Impact

The main challenge of the BioTechCOACH-ForALL project is changing attitudes of older age HF patients toward a more active lifestyle through an e-coaching system. To achieve this scope, the project implements two studies, a lab experimental study and an at-home staggered 3-phase pilot study, the former being a preparatory step for the realization of the latter. Thorough clinical evaluation preceding each study and phase will ensure patients safety. Lab study will explore the design elements (visual appearance) of the virtual agent that make it more engaging to the patient and will allow the choice of the most suitable ones for implementation of the e-coaching intervention during home study. Home study phase 1 will provide valuable information on patient clinical capacity and daily activity levels that will be used to build an individualized exercise program to be used in home study phases 2 and 3. The third and most challenging phase (home study) of the described protocol will implement an e-coaching system to provide personalized recommendations received by the patient in the comfort of their home. Main innovation points of the envisioned e-coaching technology implementation and evaluation include (1) radical new human-computer interaction paradigms through projected content on home surfaces, (2) neuroscience-backed design of virtual agents as coaches for the patients, and (3) large-scale analytics of continuous, real-life outcome metrics passively generated by patients.

### Strengths and Limitations

An important strength of this study is the fact that is the first to examine the potential of neuroscience-backed e-coaching toward patient activation and adoption of active lifestyle by HF patients. There is currently a significant gap with respect to the adoption and use adherence by chronic patients of eHealth interventions delivered at home.

This study also has some limitations. As heart failure with reduced ejection fraction is more common in men than in women (who in turn are more susceptible to heart failure with preserved ejection fraction), the percentage of male participants is expected to outrange that of females. So far, only male participants have accepted and started the study. In addition, as the inclusion criteria indicate, only patients with adequate level of technology proficiency can participate in the study. This fact complicates the generalizability of the results for older HF patients and women in particular.

The second limitation is that the nature of this study (single-case series) does not allow for a distinct control group. Each case will serve as both control and intervention participant, and analysis will be performed on an individual basis. However, one of the main strengths of this evaluation design is its real-life nature and that any validity threats will be mitigated by detailing the context and participants when results are reported.

## Appendix

Multimedia Appendix 1 Peer-reviewer report from the funding agency.

## Abbreviations

EEG: electroencephalogram
HF: heart failure
NYHA: New York Heart Association
