# Supplementary material for: Development of Cognitive and Physical Exercise Systems, Clinical Recordings, Large-Scale Data Analytics, and Virtual Coaching for Heart Failure Patients: Protocol for the BioTechCOACH-ForALL Project
Source: JMIR Res Protoc. 2020 May 4;9(5):e17714. doi: 10.2196/17714 (PMC7235814; doi:10.2196/17714)
Supplement: Multimedia Appendix 1 [file resprot_v9i5e17714_app1.pdf]

ΑΡΙΣΤΟΤΕΛΕΙΟ ΠΑΝΕΠΙΣΤΗΜΙΟ ΘΕΣΣΑΛΟΝΙΚΗΣ
ΣΧΟΛΗ ΕΠΙΣΤΗΜΩΝ ΥΓΕΙΑΣ
ΤΜΗΜΑ ΙΑΤΡΙΚΗΣ

ARISTOTLE UNIVERSITY OF THESSALONIKI
SCHOOL OF HEALTH SCIENCES
DEPARTMENT OF MEDICINE

Faculty of Health Sciences, Lab of Medical Physics

Aristotle University of Thessaloniki

P.C. 54124

Thessaloniki, Greece

January 7th, 2020

Journal of Medical Internet Research, Research Protocols

RE: "BioTechCOACH-ForALL: Development of Cognitive and Physical Exercise Systems, Clinical Recording, Large-scale Data analytics and Virtual Coaching of Heart Failure Patients: a Study Design"

Dear Madame/ Sir,

The above-mentioned project is funded by the Operational Program "Human Resources Development, Education and Lifelong Learning" and is co-financed by the European Union (European Social Fund) and Greek national funds. The project protocol was reviewed by independent reviewers, experts on the field and the awarded grades as well as the acceptance letter are provided in the accompanying file (please note the highlighted grades, corresponding to the proposed project).

More specifically, the evaluation process was divided into two phases:
Phase 1 : Proposal Completeness and Eligibility
Phase 2 : Proposal evaluation per group of criteria

External evaluators as well as an evaluation committee of the funding body were in charge of the evaluation of the proposals. In particular, the submitted proposals were classified by scientific field, as stated by the beneficiary and was described in the call for proposals. The External reviewers proceeded to documented scoring of the proposal in relation to the following approved criteria.

The criteria were categorized into two groups:

A) Completeness and clarity of the proposal content:
1.1(a) Completeness and clarity (30%), 1.4(a) Timeline (15%) 1.5(a)Research Group Composition (20%), 1.6(a) distribution of the roles of the Research Team (10%), 1.7(a) Profile of Principal Investigator (10%).

B) Purpose
3.5(a) Innovation of the proposal (15%)

The "BioTechCOACH-ForALL: Development of Cognitive and Physical Exercise Systems, Clinical Recording, Large-scale Data analytics and Virtual Coaching of Heart Failure Patients: a Study Design" proposal (MIS 5005517) scored **96,50**.

The results of the external evaluators (a and b) were described in the following table:

**MIS**	**TOTAL SCORE**	**1.1(a)**	**1.1(b)**	**1.4(a)**	**1.4(b)**	**1.5(a)**	**1.5(b)**	**1.6(a)**	**1.6(b)**	**1.7(a)**	**1.7(b)**	**3.5(a)**	**3.5(b)**
**5005517**	96,5	100	92	100	100	100	90	100	95	100	100	95	91

The official document of this Grant Funding was published by Ministry of Development & Investments to the diavgeia.gov.gr with the number ΑΔΑ: 9Ε89465ΧΙ8 - ΓΘΦ

Yours sincerely,

Panagiotis Bamidis
Principal Investigator
